# Multimodal clinical, sleep, and autonomic correlates across the depressive spectrum: a cross-sectional study

**DOI:** 10.3389/fpsyg.2026.1833260

**Published:** 2026-06-24

**Authors:** Di Jiang, Weijing Chen, Nuo Qin, Lijun Li, Shuo Wang, Ning Zhang, Jin Huang, Jian He, Chunxue Wang

**Affiliations:** 1 Beijing Tiantan Hospital, Capital Medical University, Beijing, China; 2 Institute of Software, Chinese Academy of Sciences, Beijing, China; 3 College of Computer Science and Technology, Beijing University of Technology, Beijing, China

**Keywords:** heart rate variability, major depressive disorder, multimodal assessment, polysomnography, subthreshold depression

## Abstract

Depression is a heterogeneous condition characterized by affective symptoms, sleep disturbance, and altered physiological regulation, but the relative contribution of subjective and objective measures to depressive status remains unclear. In this cross-sectional study, 101 participants were categorized as healthy controls (HC, *n* = 47), subthreshold depression (SD, *n* = 30), and major depressive disorder (MDD, *n* = 24). Demographic characteristics, symptom questionnaires, polysomnographic variables, heart rate variability (HRV) indices, and eye-tracking measures were collected. Group differences were assessed across the three groups, and classification analyses were then performed by defining HC as the non-depressive group and combining SD and MDD as the depressive group. Univariate and multivariable logistic regression analyses were used to identify independent correlates of depressive status, and model discrimination was evaluated using the area under the receiver operating characteristic curve, accuracy, sensitivity, specificity, F1 score, and Brier score. Significant between-group differences were found for HAMD-17, PHQ-9, GAD-7, PSQI, ISI, NDQ, sleep latency, rapid eye movement latency, and RMSSD. After adjustment for age and sex, higher GAD-7 scores, greater NDQ scores, and longer rapid eye movement latency remained independently associated with depressive status. In model comparison analyses, the combined model showed the highest discriminative performance (AUC = 0.942), followed closely by the clinical model (AUC = 0.930), whereas the polysomnographic and HRV models showed lower performance. These findings indicate that depressive status in this cohort was primarily characterized by greater affective and sleep-related symptom burden, while rapid eye movement latency provided additional objective information beyond questionnaire-based assessment. However, these models should be interpreted as cohort-internal, screening-oriented discrimination models within a cross-sectional design rather than prospective, diagnostic, or etiological models.

## 1 Introduction

Depression is one of the leading contributors to disability worldwide and remains a major public health challenge (O’Leary, 2021). In clinical practice, depressive status is commonly evaluated on the basis of symptom severity, functional impairment, and structured or semi-structured clinical assessment (Zimmerman, 2012). However, depression is also a highly heterogeneous condition, with substantial variation in emotional symptoms, sleep disturbance, physiological dysregulation, and cognitive-behavioral performance (Palagini et al., 2013; Kreppke et al., 2026). This heterogeneity limits the precision of symptom-based characterization alone and has increased interest in identifying objective and quantitative markers that may complement conventional clinical assessment. Similar concerns have been raised in other psychiatric biomarker studies, where heterogeneity in clinical presentation has motivated the search for more objective indicators of disease-related dysfunction.

In addition to symptom burden, depression has also been linked to impairment in instrumental activities of daily living across the lifespan, including domains such as financial decision-making, medication management, and independent functioning (Sheehan et al., 2017; Bingham et al., 2018). These effects may be particularly relevant in middle-aged and older adults, in whom affective symptoms may coexist with cognitive, sleep-related, and functional changes. This broader functional context further supports the need for multimodal approaches to depressive characterization.

Subthreshold depression (SD), although not meeting full diagnostic criteria for major depressive disorder (MDD), is clinically meaningful because it is associated with functional impairment, elevated future risk of major depression, and substantial healthcare burden (Kroenke, 2017; Zhao et al., 2024). This makes it important to evaluate not only MDD, but also SD when examining multimodal correlates of depressive status.

Sleep disturbance is one of the most prevalent features associated with depression (Crouse et al., 2021; Sun et al., 2022). Subjective manifestations including poor sleep quality, insomnia, and nightmares, are frequently reported, while objective abnormalities in sleep continuity and sleep architecture have also been described in polysomnographic studies (Solelhac et al., 2023; Sarzetto et al., 2026). In particular, sleep latency and rapid eye movement latency may reflect changes in sleep timing and sleep-state regulation relevant to affective dysregulation (Palagini et al., 2013). Thus, sleep-related measures provide a natural bridge between clinical symptom burden and objective physiological characterization.

Beyond sleep, autonomic and behavioral measures may provide additional information about depressive status. HRV has been proposed as a marker of autonomic regulation in depression, although findings have been heterogeneous across studies (Huang et al., 2018; Kemp et al., 2014). Eye-tracking measures, including indices of inhibitory control, attentional allocation, and psychomotor performance, have also shown promise as objective markers in psychiatric research (Stolicyn et al., 2022). However, as highlighted in recent eye-movement studies, objective behavioral measures may reflect specific cognitive-control or neurofunctional dimensions rather than serving as universally robust diagnostic signatures. Consequently, their value may depend on the clinical context, the population under study, and whether they are evaluated alone or together with other modalities. Prior work has suggested that eye-movement abnormalities in depression may involve psychomotor slowing, altered attentional allocation, impaired inhibitory control, and abnormal processing of emotionally salient stimuli (Gao et al., 2023; Carvalho et al., 2015). However, these findings are not entirely consistent across paradigms, and eye-tracking measures may reflect specific neurocognitive dimensions rather than provide a broadly stable diagnostic signature.

Despite growing interest in multimodal depression assessment, evidence remains heterogeneous regarding the relative contribution of symptom-based, sleep-related, autonomic, and behavioral measures (Zhang et al., 2024; Maglanoc et al., 2020). Prior studies have often focused on single-modality markers, digital phenotyping, or specific case–control comparisons, whereas fewer studies have evaluated these domains within the same analytical framework. This is particularly relevant for conditions spanning healthy controls (HC), SD, and MDD, where the clinical gradient is meaningful but the incremental value of objective markers beyond symptom-based measures remains insufficiently defined.

Therefore, this cross-sectional study had two aims. First, we sought to characterize differences in key clinical, sleep-related, autonomic, and eye-tracking variables across HC, SD, and MDD. Second, we aimed to identify independent factors associated with depressive status and to compare the discriminative performance of clinical, polysomnographic, HRV, and combined models. We hypothesized that symptom burden and subjective sleep disturbance would show clear gradients across the three groups, that selected sleep-related objective indices would provide additional information beyond questionnaire-based assessment, and that a combined model would show the highest overall discriminative performance.

To improve alignment between the descriptive and model-based discrimination components of the study, the three-group comparisons were used to characterize the depressive spectrum, whereas the binary modeling framework was selected as the primary classification framework because of sample size considerations and pragmatic screening-oriented clinical applicability. Accordingly, the binary models should be interpreted as cohort-internal discrimination models rather than diagnostic or etiological models, and ordinal modeling was used as a sensitivity analysis to test directional consistency across the depressive spectrum.

## 2 Materials and methods

### 2.1 Study design and participants

This was a cross-sectional study conducted at Beijing Tiantan Hospital. Participants were recruited from the sleep center and psychiatry clinic of Beijing Tiantan Hospital between November 2024 and July 2025. A total of 123 individuals were initially screened for eligibility. Eligibility was determined through clinical evaluation, questionnaire screening, and availability of multimodal assessment data. After exclusion of participants with unavailable data or missing HRV data and/or polysomnography (PSG) and/or eye-tracking data, 101 participants were included in the final cross-sectional analysis. According to diagnostic grouping, participants were categorized as HC (*n* = 47), SD (*n* = 30), or MDD (*n* = 24).

The MDD group comprised participants with a clinician-confirmed diagnosis of major depressive disorder according to standard diagnostic criteria (DSM-5).

The SD group included participants who did not meet full diagnostic criteria for MDD but showed clinically relevant depressive symptoms. In the present study, SD was operationalized as the presence of 2 to 4 depressive criterion symptoms for at least 2 weeks, including at least one core symptom (depressed mood or anhedonia), based on published clinical descriptions of subthreshold depression (Kroenke, 2017). Diagnostic grouping was determined by clinical evaluation, and HAMD-17 was used to quantify symptom burden rather than serve as the sole basis for classification.

The HC group had no current psychiatric diagnosis and minimal depressive symptoms.

**Figure 1:**
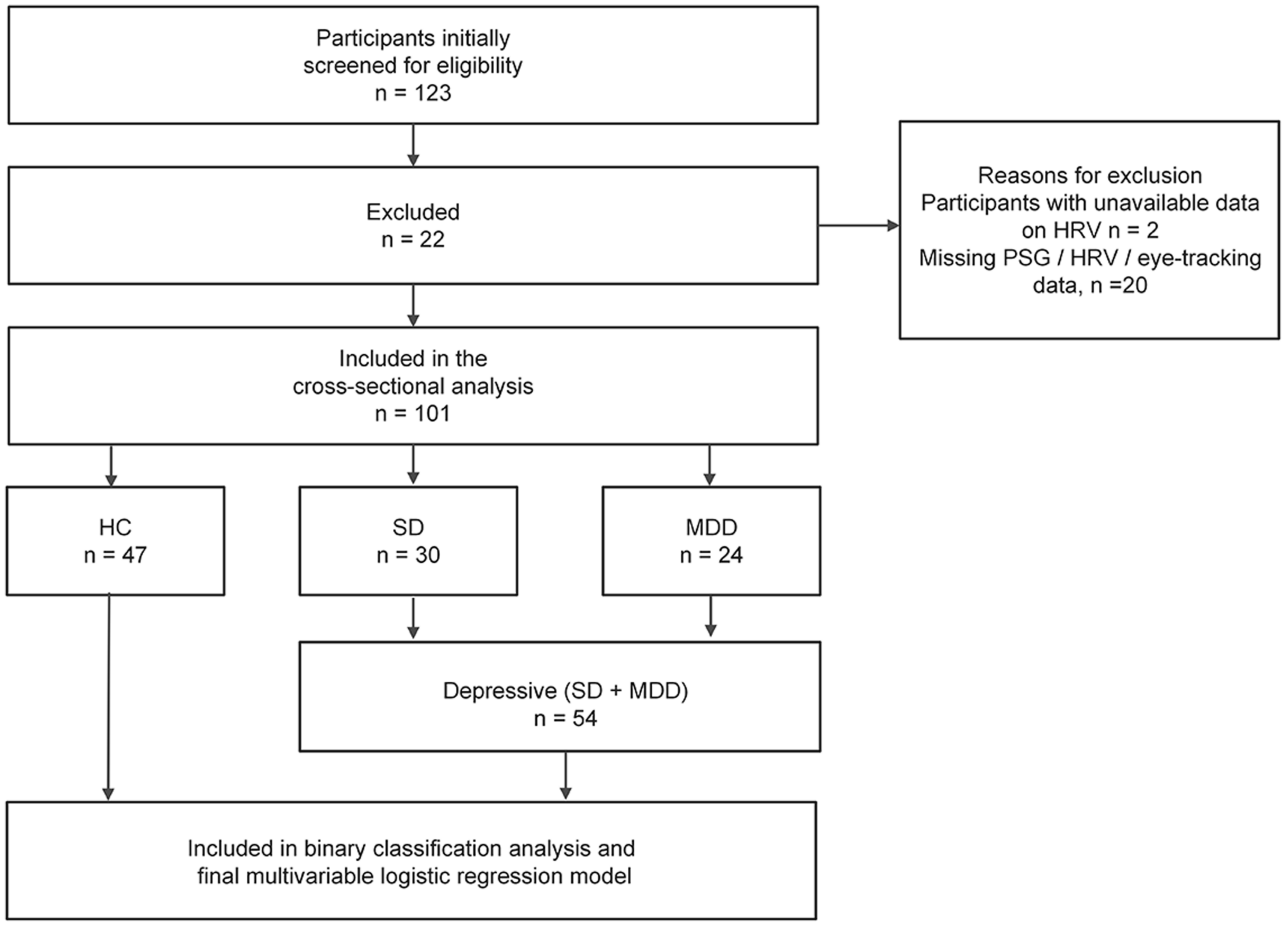
Flowchart of participant selection and data availability.

To ensure clarity of classification, diagnostic status was determined independently of symptom severity thresholds, and HAMD-17 scores were used to operationalize symptom burden in non-MDD participants. For binary classification analyses, HC participants were defined as the non-depressive group, whereas SD and MDD participants were combined into the depressive group. The participant flow and data availability are shown in Figure 1. Because this was an exploratory cross-sectional study based on consecutively recruited participants undergoing multimodal assessment, the sample size was determined pragmatically by recruitment feasibility and data completeness rather than by a formal *a priori* sample size calculation for model development.

### 2.2 Clinical and demographic assessment

All participants completed the 17-item Hamilton Depression Rating Scale (HAMD-17), the Patient Health Questionnaire-9 (PHQ-9), the Generalized Anxiety Disorder-7 (GAD-7), the Pittsburgh Sleep Quality Index (PSQI), the Insomnia Severity Index (ISI), the Nightmare Distress Questionnaire (NDQ), and STOP-Bang. These measures were used to characterize depressive symptoms, anxiety, subjective sleep quality, insomnia severity, nightmare-related symptoms, and sleep apnea risk. Demographic data included age, sex, body mass index (BMI), ethnicity, educational attainment, and marital status. Ethnicity was categorized as Han versus non-Han. Educational attainment was dichotomized as bachelor’s degree or above versus lower educational level. Marital status was dichotomized as married versus not married.

### 2.3 PSG assessment

Overnight PSG was performed using the Compumedics Grael system (Compumedics, Australia), and recordings were reviewed and processed using Profusion Sleep 4 software. All participants underwent a standard overnight PSG examination with a recording duration of approximately 8–10 h. Sleep staging and parameter scoring were conducted according to the criteria of the American Academy of Sleep Medicine (2023).

The PSG montage included electroencephalography, electrooculography, electromyography, electrocardiography, and respiratory-related signals. Electroencephalography channels included F3-M2, F4-M1, C3-M2, C4-M1, O1-M2, and O2-M1. Electrooculography channels included E1-M2 and E2-M2, and submental electromyography was recorded using a standard chin configuration. Additional signals included electrocardiography for rhythm-related monitoring, and in some recordings, respiratory parameters such as oxygen saturation, airflow, snoring, and respiratory effort were also collected.

Based on these PSG recordings, the following sleep variables were extracted for the present study. Sleep continuity parameters included total sleep time, sleep latency, wake after sleep onset, and sleep efficiency, which were used to reflect overall sleep maintenance and sleep quality. Sleep architecture parameters included the proportion and duration of N1, N2, N3, and rapid eye movement sleep, which were used to characterize overnight sleep-stage composition. Sleep fragmentation was indexed by the arousal index, and respiratory disturbance burden was quantified using the apnea-hypopnea index. These PSG-derived variables were used both to describe macro-sleep structure and to provide the physiological basis for subsequent multimodal analyses.

### 2.4 Wearable heart rate and HRV assessment

Continuous HRV monitoring was performed using the Huawei Band P8 wearable device (Huawei Technologies Co., Ltd., China). This device supports long-term, noninvasive heart rate and beat interval acquisition based on photoplethysmography and has been reported to show acceptable feasibility and stability for heart rate monitoring in resting and daily-life conditions, making it suitable for HRV-related analyses (Bellenger et al., 2021).

For each participant, HRV data were summarized from a continuous 6–10-day monitoring window with sufficient wear time and signal quality to represent typical physiological status during the baseline assessment period. Device firmware provided a signal quality index (SQI) for each segment, and only segments with SQI ≥ 80% were retained for analysis. Segments not meeting this criterion were excluded before feature calculation. RRI data then underwent artifact detection and correction, including the removal of physiologically implausible intervals and interpolation of ectopic beats. Participants without sufficient valid segments after quality filtering were not retained for complete-case multimodal analysis. Standard time-domain HRV measures were computed in accordance with guidelines from the Task Force of the European Society of Cardiology and the North American Society of Pacing and Electrophysiology, including the standard deviation of NN intervals (SDNN), the root mean square of successive differences (RMSSD), and the percentage of adjacent NN intervals differing by more than 50 ms (pNN50). Mean heart rate was also calculated from the RRI series. For the primary analyses, nighttime averages were used to reduce circadian variability. For each participant, HRV metrics were averaged across valid recording nights within the selected 6–10-day window after artifact removal and quality filtering, so that the reported values reflected cross-day summary measures.

PSG variables were derived from a single overnight laboratory recording, whereas wearable HRV metrics represented cross-day nighttime summary measures within a continuous 6–10-day monitoring window. Therefore, HRV and PSG were treated as complementary measures of physiological status rather than temporally matched signals from the same night.

### 2.5 Eye-tracking assessment

Eye-tracking data were collected using the EyeKnow intelligent eye-movement assessment system (Beijing CAS-Ruiyi Information Technology Co., Ltd., China). The system is based on the pupil center-corneal reflection method and operates at a sampling frequency of 120 Hz. Data were processed automatically using the embedded analysis algorithms provided by the system. Testing was conducted with a head-mounted display in a stable lighting environment. Before formal assessment, a standard five-point calibration procedure (up, down, left, right, and center) was performed, and calibration error was controlled within 2° to ensure measurement accuracy. After standardized instructions and demonstration, all participants completed the full test battery in approximately 10 min.

A brief battery of standardized eye-tracking tasks was administered to capture representative domains of oculomotor and cognitive-control function, including reflexive orienting, inhibitory control, response execution, and pursuit performance. For the present analyses, representative eye-tracking measures, including antisaccade accuracy, antisaccade latency, prosaccade latency, Go/No-Go accuracy, Go/No-Go latency, and smooth pursuit initiation, were included as candidate variables in multimodal modeling. Given the exploratory role of eye-tracking in the present study, these variables were treated as complementary behavioral markers rather than prespecified primary indicators of depressive status.

### 2.6 Statistical analysis and model discrimination

Continuous variables were summarized as mean ± standard deviation (SD) or median with interquartile range (IQR), as appropriate, and categorical variables as counts and percentages. For descriptive comparisons among the HC, SD, and MDD groups, continuous variables were compared using the Kruskal–Wallis test, whereas categorical variables were compared using the chi-square test or Fisher’s exact test, as appropriate. Two related but distinct analytic components were performed: an explanatory multivariable regression analysis to identify factors independently associated with depressive status, and modality-specific classification analyses to compare discriminative performance across data domains.

For the main regression analyses, depressive status was defined as SD plus MDD versus HC. Univariate logistic regression analyses were first performed to evaluate the association between each candidate variable and depressive status. Variables showing statistical relevance in univariate analyses and/or clear clinical interpretability were considered for multivariable modeling. To avoid excessive model complexity, only a limited number of representative variables were retained in the final model. Age and sex were treated as baseline covariates and were included regardless of statistical significance. Multivariable logistic regression was then used to identify independent factors associated with depressive status, and adjusted odds ratios (ORs) with 95% confidence intervals (CIs) were reported. As an additional sensitivity analysis, selected sleep-related variables were entered into an expanded model to examine the robustness of the final associations.

Candidate variables were drawn from demographic, symptom-based, sleep-related, autonomic, and eye-tracking domains. Retention of variables for the final multivariable model was based on univariate association strength, clinical interpretability, conceptual overlap with the outcome definition, and collinearity considerations. To reduce construct overlap with the depressive grouping, questionnaire measures more directly reflecting depressive symptom definition were not prioritized for the final adjusted model.

To compare discriminative performance across modalities, four logistic regression models were developed: a clinical model, a PSG model, an HRV model, and a combined multimodal model. To ensure direct comparability, all models were constructed and evaluated using the same complete-case sample, with age and sex included as baseline covariates in each model. Model performance was assessed using 5-fold cross-validation. Discrimination was evaluated by the area under the receiver operating characteristic curve (AUC), and additional performance metrics included accuracy, sensitivity, specificity, F1 score, and Brier score. The final combined model included seven variables: age, sex, GAD-7, PSQI, NDQ, REM latency, and RMSSD. Given the modest sample size, this model was intended to provide a cohort-internal estimate of multimodal discrimination rather than a definitive diagnostic model.

Cross-validation was implemented using stratified 5-fold splits. Continuous predictors were processed within the modeling pipeline, including imputation and standardization, to reduce information leakage. Classification metrics were calculated using a probability threshold of 0.5. Because final model specification was informed by the available dataset, performance estimates should be interpreted as internally validated but potentially optimistic.

Correlation analyses among selected clinical, sleep-related, and HRV variables were conducted using Spearman correlation coefficients and visualized as a heatmap in Supplementary Figure S1. As a further sensitivity analysis, ordinal logistic regression was performed using ordered diagnostic categories (HC < SD < MDD) to examine whether the main findings remained consistent when depressive status was modeled as a three-level ordered outcome. We repeated the final multivariable logistic regression after excluding participants who reported regular medication use for mood or sleep at the time of assessment.

### 2.7 Ethics statement

This study was approved by the Ethics Committee of Beijing Tiantan Hospital, Capital Medical University (KY2024-412-01). Written informed consent was obtained from all participants prior to study enrollment.

## 3 Results

### 3.1 Sample characteristics

A total of 101 participants were included in the final cross-sectional analysis, including 47 HC participants, 30 individuals with SD, and 24 patients with MDD (Figure 1). Demographic and clinical characteristics across the three groups are summarized in Table 1. There were no significant between-group differences in age, BMI, ethnicity, educational attainment, marital status, total sleep time, sleep efficiency, N2 sleep percentage, N3 sleep percentage, arousal index, or AHI. Female sex differed significantly across groups. In contrast, strong between-group differences were observed for HAMD-17, PHQ-9, GAD-7, PSQI, ISI, NDQ, sleep latency, REM latency, and RMSSD. Key differences included HAMD-17 scores of 3.30 ± 2.40 in HC, 11.33 ± 3.67 in SD, and 19.54 ± 7.98 in MDD (*p* < 0.001), as well as REM latency values of 93.59 ± 58.38, 132.53 ± 65.20, and 174.25 ± 88.30 min, respectively (*p* < 0.001).

**Table 1:** Baseline characteristics of the study population across groups.

Variable	Total (*n* = 101)	HC (*n* = 47)	SD (*n* = 30)	MDD (*n* = 24)	*p-*value
Age, year	42.43 ± 12.78	45.28 ± 14.29	39.73 ± 11.01	40.04 ± 10.04	0.139
Female, *n* (%)	66 (65.3%)	25 (53.2%)	23 (76.7%)	18 (75.0%)	0.036
BMI, kg/m^2^	23.82 ± 3.59	23.94 ± 3.18	23.76 ± 3.82	23.61 ± 4.75	0.456
Married, *n* (%)	74 (73.3%)	36 (76.6%)	21 (70.0%)	17 (70.8%)	0.778
Han ethnicity, *n* (%)	93 (92.1%)	42 (89.4%)	29 (96.7%)	22 (91.7%)	0.510
Bachelor’s degree or above, *n* (%)	58 (57.4%)	29 (61.7%)	14 (46.7%)	15 (62.5%)	0.363
Clinical scale measures					
HAMD-17 score	9.54 ± 8.03	3.30 ± 2.40	11.33 ± 3.67	19.54 ± 7.98	<0.001
PHQ-9 score	6.50 ± 6.16	1.98 ± 2.45	8.73 ± 3.34	13.38 ± 5.93	<0.001
GAD-7 score	6.01 ± 5.96	1.81 ± 2.13	7.87 ± 4.25	11.79 ± 6.19	<0.001
PSQI score	9.42 ± 5.19	5.81 ± 3.21	10.83 ± 4.05	14.71 ± 3.16	<0.001
ISI score	9.36 ± 6.84	4.79 ± 4.13	10.93 ± 6.44	15.12 ± 5.67	<0.001
NDQ score	21.33 ± 10.00	15.87 ± 5.49	24.27 ± 8.95	28.62 ± 13.04	<0.001
STOP-Bang score	1.97 ± 1.60	1.81 ± 1.66	2.00 ± 1.41	2.21 ± 1.74	0.532
Polysomnographic variables					
Total sleep time, min	409.03 ± 73.62	419.67 ± 71.89	415.12 ± 65.80	388.88 ± 89.30	0.408
Sleep efficiency, %	79.82 ± 17.29	81.58 ± 16.85	80.08 ± 19.74	78.02 ± 14.93	0.383
Sleep latency, min	22.18 ± 27.08	12.86 ± 15.34	21.40 ± 24.64	35.81 ± 35.53	0.012
REM latency, min	124.81 ± 75.22	93.59 ± 58.38	132.53 ± 65.20	174.25 ± 88.30	<0.001
N2 sleep, %	51.20 ± 14.12	49.37 ± 13.88	49.83 ± 11.29	55.15 ± 16.17	0.381
N3 sleep, %	16.57 ± 10.98	16.55 ± 9.61	15.56 ± 10.31	17.90 ± 15.20	0.945
Arousal index, events/h	14.37 ± 8.73	14.88 ± 8.23	14.44 ± 8.02	13.72 ± 10.42	0.789
AHI, events/h	9.61 ± 13.66	12.26 ± 15.35	9.49 ± 13.11	6.55 ± 12.46	0.233
HRV variables					
SDNN, ms	177.52 ± 26.98	183.81 ± 30.25	176.66 ± 23.92	166.84 ± 21.12	0.055
RMSSD, ms	200.59 ± 32.81	209.33 ± 38.74	197.57 ± 23.64	186.28 ± 26.48	0.026
pNN50, %	39.86 ± 8.92	40.88 ± 10.11	40.40 ± 6.89	38.05 ± 8.44	0.621
Eye-tracking variables					
Smooth pursuit initiation, ms	1057.15 ± 2602.71	322.08 ± 503.05	1027.56 ± 2198.34	1792.85 ± 3155.23	0.010
Antisaccade accuracy, %	55.82 ± 30.70	53.72 ± 34.92	57.08 ± 29.17	60.94 ± 22.82	0.713
Antisaccade latency, ms	300.00 ± 82.33	287.01 ± 72.98	312.24 ± 84.35	306.24 ± 94.79	0.514
Prosaccade latency, ms	270.54 ± 47.22	268.81 ± 34.14	272.74 ± 48.63	270.28 ± 70.85	0.642
Go/NoGo accuracy, %	68.00 ± 20.79	68.30 ± 22.16	67.59 ± 18.92	67.92 ± 21.26	0.932

HC, healthy controls; SD, subthreshold depression; MDD, major depressive disorder; BMI, body mass index; HAMD-17, 17-item Hamilton Depression Rating Scale; PHQ-9, Patient Health Questionnaire-9; GAD-7, Generalized Anxiety Disorder-7; PSQI, Pittsburgh Sleep Quality Index; ISI, Insomnia Severity Index; NDQ, Nightmare Disorder Questionnaire; AHI, apnea-hypopnea index; SDNN, standard deviation of normal-to-normal intervals; RMSSD, root mean square of successive differences.

### 3.2 Group differences across HC, SD and MDD

**Figure 2:**
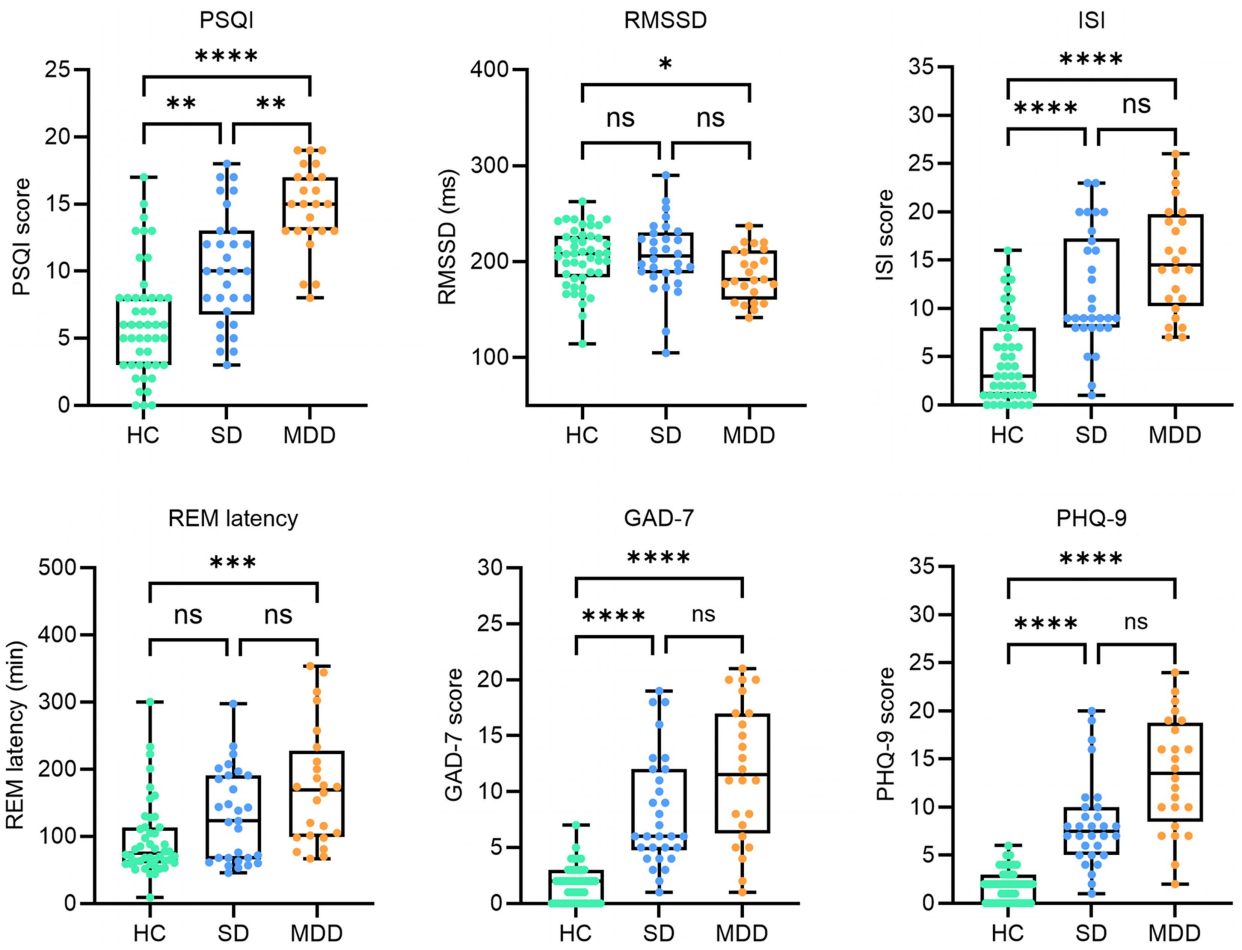
Group differences in representative questionnaire-based, sleep-related, and autonomic measures across the HC, SD, and MDD groups. Panels show PHQ-9, GAD-7, PSQI, ISI, REM latency, and RMSSD. Boxplots with individual data points are displayed. Group differences were assessed using the Kruskal–Wallis test followed by Dunn’s multiple-comparison test. *p < 0.05; **p < 0.01; ***p < 0.001; ****p < 0.0001; ns, not significant. HC, healthy controls; SD, subthreshold depression; MDD, major depressive disorder.

As shown in Table 1, Figure 2, significant between-group differences were observed in key questionnaire-based, sleep-related, and autonomic measures. HAMD-17, PHQ-9, GAD-7, PSQI, ISI, and NDQ all showed progressive worsening from HC to SD to MDD. Among PSG-derived measures, both sleep latency and REM latency were prolonged in the more symptomatic groups, with REM latency showing a particularly clear group gradient. RMSSD showed a weaker pattern of between-group difference than the questionnaire-based variables and REM latency. Overall, these findings indicate that depressive symptom burden, anxiety burden, subjective sleep disturbance, insomnia severity, and nightmare-related symptoms followed a clear diagnostic gradient, whereas objective physiological measures showed more selective between-group differences. The most prominent gradients were observed for PHQ-9, GAD-7, PSQI, ISI, and REM latency, with HC generally showing the lowest values and MDD the highest, while SD occupied an intermediate position consistent with a depressive-spectrum pattern.

### 3.3 Univariate associations with depressive status

Univariate logistic regression analyses are presented in Table 2. Higher PHQ-9 (OR = 2.546, 95% CI: 1.730–3.747), GAD-7 (OR = 2.430, 95% CI: 1.667–3.544), PSQI (OR = 1.350, 95% CI: 1.200–1.519), and NDQ scores (OR = 1.147, 95% CI: 1.070–1.229) were all significantly associated with depressive status (*p* < 0.001). Among PSG variables, REM latency showed a significant positive association (OR = 1.012 per min, 95% CI: 1.005–1.019, *p* < 0.001), whereas sleep latency showed a weaker association that did not reach the same level of statistical strength. HRV and eye-tracking variables showed comparatively weaker and less consistent associations. These results suggested that symptom burden and selected sleep timing variables were the most relevant candidate variables for subsequent multivariable analysis.

**Table 2:** Univariate logistic regression analyses for factors associated with depressive status.

Variable	*N*	OR	95% CI	*p-*value
Age, years	101	0.968	0.937–0.999	0.044
Female sex	101	0.556	0.240–1.286	0.170
PHQ-9 score	101	2.546	1.730–3.747	<0.001
GAD-7 score	101	2.430	1.667–3.544	<0.001
PSQI score	101	1.350	1.200–1.519	<0.001
ISI score	101	1.352	1.202–1.521	<0.001
NDQ score	101	1.147	1.070–1.229	<0.001
Sleep latency, min	101	1.016	0.999–1.033	0.070
REM latency, min	101	1.012	1.005–1.019	<0.001
RMSSD, ms	101	0.993	0.981–1.005	0.264
Mean night HR, bpm	101	1.038	0.988–1.091	0.138
Antisaccade accuracy, %	101	1.005	0.992–1.018	0.421

Univariate logistic regression analyses were performed to examine the association between each variable and depressive status. Depressive status was defined as subthreshold depression plus major depressive disorder versus healthy controls. Odds ratios (ORs) with 95% confidence intervals (CIs) are reported.

### 3.4 Independent factors associated with depressive status

Table 3 presents the final adjusted multivariable logistic regression model, which included age, sex, GAD-7, NDQ, and REM latency. After adjustment for age and sex, higher GAD-7 scores, greater NDQ scores, and longer REM latency remained independently associated with depressive status, whereas age and sex themselves were not statistically significant. Specifically, GAD-7 remained the strongest independent correlate (adjusted OR = 2.560, 95% CI: 1.545–4.241, p < 0.001), followed by REM latency (adjusted OR = 1.016 per min, 95% CI: 1.004–1.027, *p* = 0.009) and NDQ (adjusted OR = 1.146, 95% CI: 1.001–1.313, *p* = 0.049). This association should be interpreted cautiously because REM architecture may also be influenced by medication exposure, sleep comorbidity, and sampling context.

**Table 3:** Multivariable logistic regression model for depressive status.

Variable	Adjusted OR	95% CI	*p*-value
Age, years	1.002	0.943–1.066	0.938
Female sex	0.422	0.087–2.042	0.283
GAD-7 score	2.560	1.545–4.241	<0.001
NDQ score	1.146	1.001–1.313	0.049
REM latency, min	1.016	1.004–1.027	0.009

Multivariable logistic regression analysis was performed to identify independent factors associated with depressive status. The model was adjusted for age and sex. Depressive status was defined as subthreshold depression plus major depressive disorder versus healthy controls. Odds ratios (ORs) with 95% confidence intervals (CIs) are reported. The final model was based on participants with complete data for the included variables.

As a sensitivity analysis, we repeated the final multivariable logistic regression after excluding participants who reported regular medication use for mood or sleep at the time of assessment (Supplementary Table S2). In this restricted sample, higher GAD-7 scores remained significantly associated with depressive status (adjusted OR = 2.850, 95% CI: 1.297–6.262, p = 0.009), whereas NDQ and REM latency showed directionally consistent but attenuated associations (adjusted OR = 1.229, 95% CI: 0.985–1.533, *p* = 0.068; adjusted OR = 1.020, 95% CI: 0.999–1.041, *p* = 0.059, respectively).

### 3.5 Discriminative performance of unimodal and multimodal models

**Figure 3:**
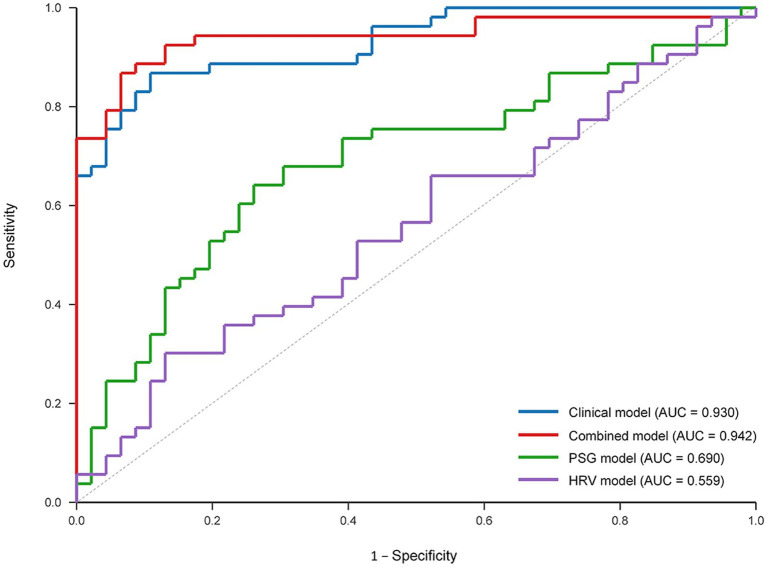
ROC curves of modality-specific and combined discrimination models for depressive status (SD and MDD vs. HC). Clinical model: age, sex, GAD-7, PSQI, ISI, NDQ; PSG model: age, sex, TST, sleep efficiency, sleep latency, REM latency, N2%, N3%, AHI, arousal index; HRV model: age, sex, mean day HR, mean night HR, Mean RRI, SDNN, RMSSD, pNN50; Combined model: age, sex, GAD-7, PSQI, NDQ, REM latency, RMSSD. All models were developed and evaluated on the same complete-case sample using stratified 5-fold cross-validation.

The discriminative performance of the clinical, PSG, HRV, and combined models is summarized in Table 4 and illustrated in Figure 3. The combined model showed the highest discriminative performance (AUC = 0.942, F1 score = 0.891), followed closely by the clinical model (AUC = 0.930, F1 score = 0.874). In contrast, the PSG model showed only moderate discrimination, whereas the HRV model showed limited utility for discrimination in this cohort. The relatively small difference between the combined and clinical models suggests that questionnaire-based measures captured most of the discriminative information in this cohort. Nevertheless, the inclusion of selected objective variables, particularly REM latency, provided modest additional value. Notably, the difference between the combined and clinical models was small, whereas both outperformed the PSG and HRV models by a wider margin, supporting the interpretation that symptom-based measures accounted for most of the discrimination in this cohort.

**Table 4:** Discriminative performance of different models for depressive status.

Model	AUC (95% CI)	Accuracy	Sensitivity	Specificity	F1 score	Brier score
Clinical model	0.930 (0.876–0.972)	0.869	0.849	0.891	0.874	0.104
PSG model	0.690 (0.579–0.787)	0.677	0.679	0.674	0.692	0.227
HRV model	0.559 (0.446–0.672)	0.535	0.585	0.478	0.574	0.258
Combined multimodal model	0.942 (0.887–0.980)	0.889	0.849	0.935	0.891	0.087

Discriminative performance was evaluated using the area under the receiver operating characteristic curve (AUC), accuracy, sensitivity, specificity, F1 score, and Brier score. All models were developed and evaluated on the same complete-case sample, with age and sex included as baseline covariates. Performance was assessed using stratified 5-fold cross-validation, and AUC confidence intervals were estimated by bootstrap resampling.

### 3.6 Correlation analysis

As a sensitivity analysis, ordinal logistic regression using ordered diagnostic categories (HC < SD < MDD) showed that higher GAD-7 scores, greater NDQ scores, and longer REM latency remained significantly associated with increasing depressive severity (Supplementary Table S1), supporting the directional consistency of the main findings across the depressive spectrum.

Spearman correlation analysis among selected clinical, sleep-related, and HRV variables is shown in Supplementary Figure S1. Questionnaire-based symptom measures, including HAMD-17, PHQ-9, GAD-7, PSQI, ISI, and NDQ, were moderately to strongly intercorrelated. Sleep latency and REM latency showed positive correlations with several symptom-based measures, although these relationships were generally weaker than those observed among the questionnaire variables themselves. By contrast, HRV indices, including RMSSD, SDNN, and pNN50, showed comparatively weaker and less consistent correlations with symptom and sleep variables.

## 4 Discussion

In this cross-sectional study, we examined multimodal correlates of depressive status by integrating questionnaire-based symptoms, PSG-derived sleep variables, HRV indices, and eye-tracking measures. Three principal findings emerged. First, questionnaire-based symptom and sleep-related measures showed clear gradients across HC, SD, and MDD. Second, after adjustment for age and sex, GAD-7 and REM latency emerged as the most consistent correlates of depressive status, while NDQ showed a weaker association that should be interpreted more cautiously. Third, although the combined model achieved the highest discriminative performance, its advantage over the clinical model was modest, indicating that questionnaire-based measures captured most of the discriminative information in this cohort.

### 4.1 Clinical gradient across HC, SD and MDD

The progressive increases in HAMD-17, PHQ-9, GAD-7, PSQI, ISI, and NDQ across the three groups support the clinical relevance of the HC-SD-MDD spectrum used in the descriptive component of the study. This pattern suggests that SD was not merely a residual or ambiguous category, but instead occupied an intermediate and clinically meaningful position between healthy status and MDD. Such a gradient is broadly consistent with previous evidence showing that subthreshold depressive states are associated with psychosocial impairment and may represent part of a dimensional continuum of depressive psychopathology rather than a fully distinct category (Judd et al., 1997; Judd et al., 2000; Noyes et al., 2022). From a clinical perspective, this supports the view that symptom burden, particularly across affective and sleep-related domains, may be more informative than diagnosis alone when characterizing early or intermediate depressive states.

### 4.2 REM latency in depression

Among the objective sleep measures, REM latency emerged as the most consistent marker in the present study. This finding is noteworthy because REM-related sleep abnormalities have long been considered one of the more characteristic physiological features associated with depression (Baglioni et al., 2016; Ricciardiello et al., 2024). Large meta-analytic studies have shown that depressive disorders are often characterized by disturbances in sleep continuity together with altered REM-related parameters, and more recent systematic reviews continue to support the relevance of REM sleep abnormalities in major depression (Arıkan et al., 2024; Zhang et al., 2023). In our cohort, REM latency remained independently associated with depressive status even after adjustment for age and sex, suggesting that objective REM-related sleep timing may capture information beyond questionnaire-based symptom burden.

At the same time, this finding should be interpreted cautiously because the direction of association in our cohort does not fully align with the classical literature emphasizing shortened REM latency in major depression. This inconsistency is not entirely unexpected. REM latency is highly sensitive to sample composition, age structure, psychiatric and sleep comorbidities, respiratory disturbance, and night-to-night variability (Leseur et al., 2025; Leitner et al., 2025; Rosenblum et al., 2025). Moreover, the literature itself is not fully uniform, and recent reviews suggest that although REM abnormalities remain conceptually important in depression, the robustness and direction of specific REM latency findings may be weaker than historically assumed once study quality and heterogeneity are taken into account. In this sense, our result may reflect the characteristics of a real-world multimodal psychiatric-sleep cohort rather than a direct replication of the classical short-REM-latency phenotype.

A further consideration is medication exposure. Some patients with major depressive disorder in the present study had already been receiving hypnotic or psychotropic treatment, which may have influenced REM architecture (Steiger and Pawlowski, 2019). In particular, many antidepressants and some sleep-promoting medications are known to suppress REM sleep and prolong REM latency, potentially weakening or even reversing the classical short-REM-latency pattern reported in highly selected drug-free samples (Wichniak et al., 2017; Rijnbeek et al., 2003). To address this issue, we performed a sensitivity analysis restricted to participants not receiving regular medication for mood or sleep. In that restricted sample, the direction of association for REM latency remained unchanged, although statistical significance was attenuated. This pattern suggests that medication exposure may have influenced the magnitude of the observed association, but is unlikely to fully account for its direction. At the same time, this sensitivity analysis should be interpreted with caution because the restricted sample was substantially smaller and included very few non-medicated MDD participants, limiting statistical power and subgroup representativeness. Taken together, these findings suggest that REM latency should be interpreted as a cohort-specific association rather than a definitive physiological signature of depression.

By contrast, broader macro-sleep parameters such as total sleep time, sleep efficiency, and stage percentages did not show similarly robust independent effects. This pattern may indicate that depressive status in this sample was more strongly linked to alterations in the temporal organization of sleep than to a global reduction in sleep quantity or a uniform shift in overall stage proportions. This interpretation is compatible with transdiagnostic sleep meta-analytic findings suggesting that REM-related and sleep-depth parameters may be more disorder-relevant or comorbidity-sensitive than sleep quantity alone (Eckert and Sweetman, 2020; Mendoza Alvarez et al., 2025).

### 4.3 HRV and eye-tracking assessment in depression

The HRV findings were more modest. RMSSD showed some degree of group-level difference, but HRV variables did not contribute strongly to the final multivariable models. This should not be interpreted as evidence against autonomic involvement in depression. On the contrary, previous meta-analyses have consistently reported lower HRV in adults with depression, including reductions in SDNN, RMSSD, and pNN50 (Koch et al., 2019; Wu et al., 2023). Nevertheless, the magnitude and stability of HRV abnormalities vary substantially across studies, partly because HRV is highly sensitive to age, medication use, cardiovascular status, physical activity, circadian phase, respiratory patterns, and psychiatric comorbidity (Wu et al., 2023; Brown et al., 2018). In the present cohort, HRV may therefore have functioned better as a complementary physiological descriptor than as a strong standalone classifier. Put differently, autonomic dysregulation may be present, but its signal appears smaller and less specific than the symptom and sleep-timing signals in this particular sample.

A similar interpretation applies to the eye-tracking findings. Although selected oculomotor variables showed some group-level variation, they did not contribute substantially to classification performance when compared with questionnaire-based variables. This is not entirely surprising. Prior reviews suggest that eye-movement abnormalities in depression, including slower prosaccade and antisaccade performance and altered processing of emotional stimuli, may reflect psychomotor slowing, inhibitory control dysfunction, or attentional bias rather than a highly specific diagnostic signature (Carvalho et al., 2015; Dotson et al., 2020; Ferguson and Cane, 2017). Therefore, in th**is** study eye-tracking is best positioned as an exploratory behavioral characterization tool rather than a central determinant of diagnostic discrimination. Our results are broadly compatible with that view.

### 4.4 Implications of multimodal modeling

One of the most clinically relevant findings is that the clinical model nearly matched the combined model in discriminative performance. This suggests that conventional symptom-based assessment remains highly informative for distinguishing depressive from non-depressive participants in similar cohorts. In practical terms, questionnaires capturing depressive symptoms, anxiety burden, insomnia, sleep quality, and nightmare-related distress already contain a large proportion of the information needed for classification. At the same time, the modest improvement seen in the combined model indicates that selected objective markers can still provide incremental value. In the present study, REM latency appears to be the most promising candidate for such an incremental role. Thus, rather than replacing symptom-based assessment, multimodal markers may be better positioned as adjunctive indicators that enrich clinical characterization.

At the same time, the interpretation of the clinical model requires caution because several symptom-based measures are conceptually close to the grouping framework used in this cohort. Therefore, the high performance of the clinical model should be understood as reflecting cohort-internal screening-oriented discrimination rather than a diagnostic model or a claim of biomarker-level specificity. In this sense, the modest incremental gain from objective markers is informative, but it should not be interpreted as evidence that physiological measures lack broader clinical relevance.

The correlation heatmap further supports this interpretation. Questionnaire-based measures were moderately to strongly intercorrelated, consistent with a clustered symptom structure encompassing depression severity, anxiety, insomnia, poor sleep quality, and nightmare burden. In contrast, objective sleep and HRV variables showed weaker and less consistent correlations with the symptom measures, suggesting that they captured partially overlapping but not identical dimensions of depressive status. This pattern reinforces the broader conclusion of the study: questionnaire-based measures dominate discrimination, whereas objective physiological variables provide selective but potentially meaningful additional information.

Beyond these modality-specific considerations, cultural context may also be relevant when interpreting symptom reporting and help-seeking behavior in depressive disorders. In particular, the expression of affective distress, sleep complaints, and nightmare-related burden may be shaped by sociocultural norms, stigma, and health-seeking patterns. These factors should be considered when interpreting questionnaire-based and behavioral findings from a single-site Chinese cohort and when assessing the generalizability of the present results to other populations.

### 4.5 Strengths and limitations

This study integrated multiple clinical and physiological domains within a single analytical framework, distinguished clearly between descriptive three-group comparison and binary discrimination modeling, and compared models on the same complete-case sample to improve fairness of performance evaluation. However, there are still several limitations. First, the sample size was modest, especially for multimodal model development and comparison, which may have limited the stability of more complex models and constrained more formal three-category modeling. Although the final adjusted model was intentionally restricted to a limited number of representative variables, the risk of overfitting cannot be fully excluded, particularly for the combined multimodal discrimination model. Second, the cross-sectional design precludes causal inference and does not allow determination of whether the observed multimodal features are state markers, trait markers, or correlates of illness burden. Third, some physiological and behavioral measures may require repeated assessments, task refinement, or larger samples to demonstrate stronger and more stable effects. Fourth, because depressive status was strongly reflected in questionnaire-based measures, the potential incremental contribution of objective markers was constrained by the already high performance of the clinical model. Fifth, medication exposure and sleep comorbidity could not be fully standardized in this real-world cohort and therefore remain important interpretive considerations, particularly for REM-related findings. Sixth, there was potential construct overlap between symptom-based questionnaire variables and the definition of depressive grouping, which may partly explain the strong performance of the clinical model. Furthermore, although cross-validation was used, the present model-based discrimination analyses remain internally validated only, and the reported performance estimates may still be somewhat optimistic in the absence of external validation or fully nested model selection. In addition, several potentially relevant factors, including body weight-related functional burden, fatigue severity, and non-psychiatric comorbid diseases, were not systematically modeled in the present analysis. These variables may influence both physiological and behavioral measures and should be incorporated in future studies. Moreover, although questionnaire-based instruments are clinically practical and informative, self-report measures should be interpreted with caution because they may not fully capture observable functional or physiological impairment without complementary objective assessment or collateral reports. Finally, the direction of the REM latency finding should be interpreted cautiously and verified in future studies with larger samples, supplementary ordinal or multinomial sensitivity analyses, stricter control of medication status and sleep comorbidity, and repeated-night PSG.

## 5 Conclusion

Depressive status in this cohort was primarily reflected by a greater affective burden and more severe subjective sleep disturbance, with a clear gradient across HC, SD, and MDD. Among the objective measures, rapid eye movement latency provided incremental information beyond questionnaire-based assessment and remained independently associated with depressive status after adjustment for age and sex. Although the combined multimodal model showed the best overall discriminative performance, its improvement over the clinical model was limited, suggesting that symptom-based measures accounted for most of the discriminative capacity in this sample. Taken together, these findings support a pragmatic multimodal framework in which clinical assessment remains the foundation, while selected objective physiological markers, particularly sleep-related measures, may provide complementary value for clinical characterization rather than serving as stand-alone substitutes for conventional evaluation.

## Data Availability

The raw data supporting the conclusions of this article will be made available by the authors, without undue reservation.

## References

[ref22] American Academy of Sleep Medicine (2023). The AASM Manual for the Scoring of Sleep and Associated Events 3.0. Darien, IL: American Academy of Sleep Medicine.

[ref1] Arıkan M. K. Uysal Ö. Gıca Ş. Orhan Ö. İlhan R. Esmeray M. T. . (2024). Rem parameters in drug-free major depressive disorder: a systematic review and meta-analysis. Sleep Med. Rev. 73:101876. doi: 10.1016/j.smrv.2023.101876, 37995418

[ref2] Baglioni C. Nanovska S. Regen W. Spiegelhalder K. Feige B. Nissen C. . (2016). Sleep and mental disorders: a meta-analysis of polysomnographic research. Psychol. Bull. 142, 969–990. doi: 10.1037/bul0000053, 27416139 PMC5110386

[ref3] Bellenger C. R. Miller D. J. Halson S. L. Roach G. D. (2021). Wrist-based photoplethysmography assessment of heart rate and heart rate variability: validation of whoop. Sensors (Basel) 21:3571. doi: 10.3390/s21103571, 34065516 PMC8160717

[ref4] Bingham K. S. Kumar S. Dawson D. R. Mulsant B. H. Flint A. J. (2018). A systematic review of the measurement of function in late-life depression. Am. J. Geriatr. Psychiatry 26, 54–72. doi: 10.1016/j.jagp.2017.08.011, 29050912

[ref5] Brown L. Karmakar C. Gray R. Jindal R. Lim T. Bryant C. (2018). Heart rate variability alterations in late life depression: a meta-analysis. J. Affect. Disord. 235, 456–466. doi: 10.1016/j.jad.2018.04.071, 29679898

[ref6] Carvalho N. Laurent E. Noiret N. Chopard G. Haffen E. Bennabi D. (2015). Eye movement in unipolar and bipolar depression: a systematic review of the literature. Front. Psychol. 6:1809. doi: 10.3389/fpsyg.2015.01809, 26696915 PMC4678228

[ref7] Crouse J. J. Carpenter J. S. Song Y. J. C. Hockey S. J. Naismith S. L. Grunstein R. R. . (2021). Circadian rhythm sleep-wake disturbances and depression in young people: implications for prevention and early intervention. Lancet Psychiatry 8, 813–823. doi: 10.1016/S2215-0366(21)00034-1, 34419186

[ref8] Dotson V. M. Mcclintock S. M. Verhaeghen P. Kim J. U. Draheim A. A. Syzmkowicz S. M. . (2020). Depression and cognitive control across the lifespan: a systematic review and Meta-analysis. Neuropsychol. Rev. 30, 461–476. doi: 10.1007/s11065-020-09436-6, 32385756 PMC9637269

[ref9] Eckert D. J. Sweetman A. (2020). Impaired central control of sleep depth propensity as a common mechanism for excessive overnight wake time: implications for sleep apnea, insomnia and beyond. J. Clin. Sleep Med. 16, 341–343. doi: 10.5664/jcsm.8268, 32003739 PMC7075088

[ref10] Ferguson H. J. Cane J. (2017). Tracking the impact of depression in a perspective-taking task. Sci. Rep. 7:14821. doi: 10.1038/s41598-017-13922-y, 29093490 PMC5666009

[ref11] Gao M. Xin R. Wang Q. Gao D. Wang J. Yu Y. (2023). Abnormal eye movement features in patients with depression: preliminary findings based on eye tracking technology. Gen. Hosp. Psychiatry 84, 25–30. doi: 10.1016/j.genhosppsych.2023.04.010, 37307718

[ref12] Huang M. Shah A. Su S. Goldberg J. Lampert R. J. Levantsevych O. M. . (2018). Association of Depressive Symptoms and Heart Rate Variability in Vietnam war-era twins: a longitudinal twin difference study. JAMA Psychiatry 75, 705–712. doi: 10.1001/jamapsychiatry.2018.0747, 29799951 PMC6059565

[ref13] Judd L. L. Akiskal H. S. Paulus M. P. (1997). The role and clinical significance of subsyndromal depressive symptoms (SSD) in unipolar major depressive disorder. J. Affect. Disord. 45, 5–17. discussion 17-8 9268771 10.1016/s0165-0327(97)00055-4

[ref14] Judd L. L. Akiskal H. S. Zeller P. J. Paulus M. Leon A. C. Maser J. D. . (2000). Psychosocial disability during the long-term course of unipolar major depressive disorder. Arch. Gen. Psychiatry 57, 375–380. doi: 10.1001/archpsyc.57.4.375, 10768699

[ref15] Kemp A. H. Brunoni A. R. Santos I. S. Nunes M. A. Dantas E. M. Carvalho De Figueiredo R. . (2014). Effects of depression, anxiety, comorbidity, and antidepressants on resting-state heart rate and its variability: an Elsa-Brasil cohort baseline study. Am. J. Psychiatry 171, 1328–1334. doi: 10.1176/appi.ajp.2014.13121605, 25158141

[ref16] Koch C. Wilhelm M. Salzmann S. Rief W. Euteneuer F. (2019). A meta-analysis of heart rate variability in major depression. Psychol. Med. 49, 1948–1957. doi: 10.1017/S0033291719001351, 31239003

[ref17] KreppkeJ. N. BrupbacherG. RupfK. HohbergV. CodyR. GerberM. . (2026). Association between physical activity, heart rate variability and major depressive disorders: an umbrella review. Sports Med. 56, 703–723. doi: 10.1007/s40279-025-02365-5, 41639538

[ref18] Kroenke K. (2017). When and how to treat subthreshold depression. JAMA 317, 702–704. doi: 10.1001/jama.2017.0233, 28241337

[ref19] Leitner C. Dalle Piagge F. Tomic T. Nozza F. Fasiello E. Castronovo V. . (2025). Sleep alterations in major depressive disorder and insomnia disorder: a network meta-analysis of polysomnographic studies. Sleep Med. Rev. 80:102048. doi: 10.1016/j.smrv.2025.102048, 40054014

[ref20] Leseur J. Maruani J. Palagini L. Lejoyeux M. Geoffroy P. A. (2025). Objective sleep markers to differentiate unipolar and bipolar depression: a systematic review and meta-analysis. Neurosci. Biobehav. Rev. 171:106070. doi: 10.1016/j.neubiorev.2025.106070, 39978428

[ref21] Maglanoc L. A. Kaufmann T. Jonassen R. Hilland E. Beck D. Landrø N. I. . (2020). Multimodal fusion of structural and functional brain imaging in depression using linked independent component analysis. Hum. Brain Mapp. 41, 241–255. doi: 10.1002/hbm.24802, 31571370 PMC7267936

[ref23] Mendoza Alvarez M. Balthasar Y. Verbraecken J. Claes L. Van Someren E. Van Marle H. J. F. . (2025). Systematic review: rem sleep, dysphoric dreams and nightmares as transdiagnostic features of psychiatric disorders with emotion dysregulation - clinical implications. Sleep Med. 127, 1–15. doi: 10.1016/j.sleep.2024.12.037, 39756154

[ref24] Noyes B. K. Munoz D. P. Khalid-Khan S. Brietzke E. Booij L. (2022). Is subthreshold depression in adolescence clinically relevant? J. Affect. Disord. 309, 123–130. doi: 10.1016/j.jad.2022.04.067, 35429521

[ref25] O’Leary K. (2021). Global increase in depression and anxiety. Nat. Med. doi: 10.1038/d41591-021-00064-y 34675435

[ref26] Palagini L. Baglioni C. Ciapparelli A. Gemignani A. Riemann D. (2013). Rem sleep dysregulation in depression: state of the art. Sleep Med. Rev. 17, 377–390. doi: 10.1016/j.smrv.2012.11.001, 23391633

[ref27] Ricciardiello A. Teh J. Z. Lam A. K. F. Marshall N. S. Naismith S. L. D'rozario A. L. (2024). Objective measures of sleep in adults and older adults with and without depression: a systematic review and meta-analysis. Sleep Med. 124, 637–648. doi: 10.1016/j.sleep.2024.10.011, 39515262

[ref28] Rijnbeek B. De Visser S. J. Franson K. L. Cohen A. F. Van Gerven J. M. (2003). Rem sleep effects as a biomarker for the effects of antidepressants in healthy volunteers. J. Psychopharmacol. 17, 196–203. doi: 10.1177/0269881103017002008, 12870567

[ref29] Rosenblum Y. Nakagawa J. Van Hattem T. Krugliakova E. Sabhapondit B. Bovy L. . (2025). Sleep neurophysiology in depression. Biol. Psychiatry 98, 842–853. doi: 10.1016/j.biopsych.2025.07.023, 40769448

[ref30] Sarzetto A. Cavallini M. C. Pacchioni F. Casoni F. Ferini Strambi L. Franchini L. . (2026). Nocturnal wakefulness is associated with suicidality in depressed inpatients: a polysomnographic study. J. Sleep Res. 35:e70138. doi: 10.1111/jsr.70138, 40624816

[ref31] Sheehan D. V. Nakagome K. Asami Y. Pappadopulos E. A. Boucher M. (2017). Restoring function in major depressive disorder: a systematic review. J. Affect. Disord. 215, 299–313. doi: 10.1016/j.jad.2017.02.029, 28364701

[ref32] Solelhac G. Berger M. Strippoli M. F. Marchi N. A. Stephan A. Petit J. M. . (2023). Objective polysomnography-based sleep features and major depressive disorder subtypes in the general population. Psychiatry Res. 324:115213. doi: 10.1016/j.psychres.2023.115213, 37098299

[ref33] Steiger A. Pawlowski M. (2019). Depression and sleep. Int. J. Mol. Sci. 20:607. doi: 10.3390/ijms20030607, 30708948 PMC6386825

[ref34] Stolicyn A. Steele J. D. Seriès P. (2022). Prediction of depression symptoms in individual subjects with face and eye movement tracking. Psychol. Med. 52, 1784–1792. doi: 10.1017/S0033291720003608, 33161920

[ref35] Sun X. Liu B. Liu S. Wu D. J. H. Wang J. Qian Y. . (2022). Sleep disturbance and psychiatric disorders: a bidirectional Mendelian randomisation study. Epidemiol. Psychiatr. Sci. 31:e26. doi: 10.1017/S2045796021000810, 35465862 PMC9069588

[ref36] Wichniak A. Wierzbicka A. Walęcka M. Jernajczyk W. (2017). Effects of antidepressants on sleep. Curr. Psychiatry Rep. 19:63. doi: 10.1007/s11920-017-0816-4, 28791566 PMC5548844

[ref37] Wu Q. Miao X. Cao Y. Chi A. Xiao T. (2023). Heart rate variability status at rest in adult depressed patients: a systematic review and meta-analysis. Front. Public Health 11:1243213. doi: 10.3389/fpubh.2023.1243213, 38169979 PMC10760642

[ref38] Zhang W. Mao K. Chen J. (2024). A multimodal approach for detection and assessment of depression using text, audio and video. Phenomics 4, 234–249. doi: 10.1007/s43657-023-00152-8, 39398421 PMC11467147

[ref39] Zhang Y. Ren R. Yang L. Zhang H. Shi Y. Vitiello M. V. . (2023). Patterns of polysomnography parameters in 27 neuropsychiatric diseases: an umbrella review. Psychol. Med. 53, 4675–4695. doi: 10.1017/S0033291722001581, 36377491

[ref40] Zhao X. Zhang L. Sáenz A. A. Zhang X. Sun J. Zhong Q. . (2024). Prevalence of subthreshold depression in older adults: a systematic review and meta-analysis. Asian J. Psychiatr. 102:104253. doi: 10.1016/j.ajp.2024.104253, 39388746

[ref41] Zimmerman M. (2012). Symptom severity and guideline-based treatment recommendations for depressed patients: implications of Dsm-5's potential recommendation of the Phq-9 as the measure of choice for depression severity. Psychother. Psychosom. 81, 329–332. doi: 10.1159/000342262, 22964496

