# Supplementary material for: Multimodal clinical, sleep, and autonomic correlates across the depressive spectrum: a cross-sectional study
Source: Front Psychol. 2026 Jun 24;17:1833260. doi: 10.3389/fpsyg.2026.1833260 (PMC13341429; doi:10.3389/fpsyg.2026.1833260)
Supplement: Supplementary file 1 [file Supplementary_file_1.DOCX]

Supplementary Material

# Supplementary Figures and Tables

## Supplementary Tables

**Supplementary Table S1. Ordinal logistic regression sensitivity analysis across healthy controls, subthreshold depression, and major depressive disorder**

| **Variable** | **Adjusted OR** | **95% CI** | **P value** |
| --- | --- | --- | --- |
| Age, years | 1.000 | 0.964–1.038 | 0.990 |
| Female sex | 0.940 | 0.366–2.419 | 0.898 |
| GAD-7 score | 1.342 | 1.194–1.509 | <0.001 |
| NDQ score | 1.075 | 1.013–1.140 | 0.018 |
| REM latency, min | 1.010 | 1.003–1.016 | 0.006 |

**Note:** Ordinal logistic regression was performed using ordered diagnostic categories (healthy control < subthreshold depression < major depressive disorder) as the dependent variable. The multivariable model included age, sex, GAD-7 score, NDQ score, and REM latency. Odds ratios (ORs) with 95% confidence intervals (CIs) are reported. ORs greater than 1 indicate higher odds of belonging to a more severe depressive category. The analysis was based on participants with complete data for the included variables (n = 101).

**Supplementary Table S2. Sensitivity analysis excluding participants receiving regular medication for mood or sleep**

| **Variable** | **Adjusted OR** | **95% CI** | **P value** |
| --- | --- | --- | --- |
| Age, years | 1.045 | 0.959–1.138 | 0.315 |
| Female sex | 0.459 | 0.047–4.494 | 0.503 |
| GAD-7 score | 2.850 | 1.297–6.262 | 0.009 |
| NDQ score | 1.229 | 0.985–1.533 | 0.068 |
| REM latency, min | 1.020 | 0.999–1.041 | 0.059 |

**Note:** Sensitivity analysis was performed after excluding participants who reported regular medication use for mood or sleep at the time of assessment. Multivariable logistic regression was adjusted for age and sex. Depressive status was defined as subthreshold depression plus major depressive disorder versus healthy controls. The analysis was based on participants with complete data for the included variables (n = 58).

## Supplementary Figures


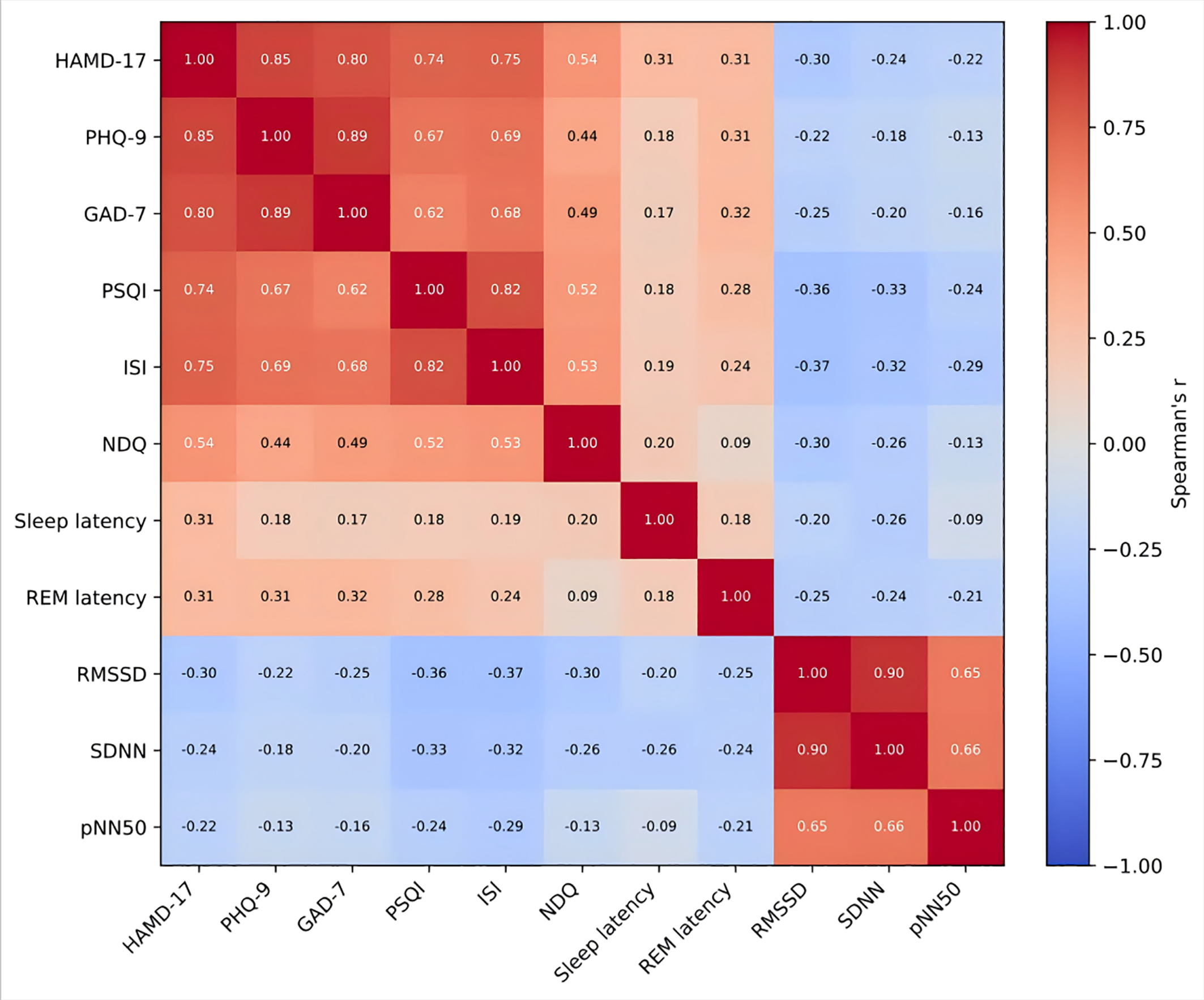


**Supplementary Figure 1. Spearman correlation heatmap of selected clinical, sleep, and heart rate variability variables.** The heatmap shows pairwise Spearman correlation coefficients among HAMD-17, PHQ-9, GAD-7, PSQI, ISI, NDQ, sleep latency, REM latency, RMSSD, SDNN, and pNN50 in participants with complete data.
